# Tetralogy of Fallot

**DOI:** 10.1186/1750-1172-4-2

**Published:** 2009-01-13

**Authors:** Frederique Bailliard, Robert H Anderson

**Affiliations:** 1 North Carolina Children's Heart Center, Department of Pediatrics, University of North Carolina at Chapel Hill, USA; 2 Division of Pediatric Cardiology, Medical University of South Carolina, Charleston, South Carolina, USA

## Abstract

Tetralogy of Fallot is a congenital cardiac malformation that consists of an interventricular communication, also known as a ventricular septal defect, obstruction of the right ventricular outflow tract, override of the ventricular septum by the aortic root, and right ventricular hypertrophy.

This combination of lesions occurs in 3 of every 10,000 live births, and accounts for 7–10% of all congenital cardiac malformations.

Patients nowadays usually present as neonates, with cyanosis of varying intensity based on the degree of obstruction to flow of blood to the lungs.

The aetiology is multifactorial, but reported associations include untreated maternal diabetes, phenylketonuria, and intake of retinoic acid. Associated chromosomal anomalies can include trisomies 21, 18, and 13, but recent experience points to the much more frequent association of microdeletions of chromosome 22. The risk of recurrence in families is 3%.

Useful diagnostic tests are the chest radiograph, electrocardiogram, and echocardiogram. The echocardiogram establishes the definitive diagnosis, and usually provides sufficient information for planning of treatment, which is surgical. Approximately half of patients are now diagnosed antenatally.

Differential diagnosis includes primary pulmonary causes of cyanosis, along with other cyanotic heart lesions, such as critical pulmonary stenosis and transposed arterial trunks. Neonates who present with ductal-dependent flow to the lungs will receive prostaglandins to maintain ductal patency until surgical intervention is performed. Initial intervention may be palliative, such as surgical creation of a systemic-to-pulmonary arterial shunt, but the trend in centres of excellence is increasingly towards neonatal complete repair. Centres that undertake neonatal palliation will perform the complete repair at the age of 4 to 6 months. Follow-up in patients born 30 years ago shows a rate of survival greater than 85%. Chronic issues that now face such adults include pulmonary regurgitation, recurrence of pulmonary stenosis, and ventricular arrhythmias. As the strategies for surgical and medical management have progressed, the morbidity and mortality of those born with tetralogy of Fallot in the current era is expected to be significantly improved.

## Background

**Figure 1:**
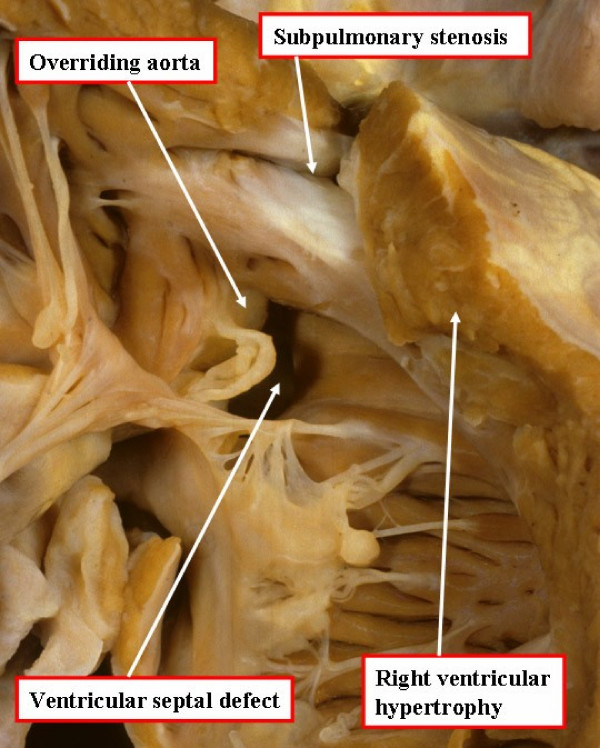
**An autopsied specimen has been opened through the anterior wall of the right ventricle to show the cardinal features of tetralogy of Fallot**.

La Maladie Bleue, as described by Louis Arthur Etienne Fallot in 1888 [1], is the clinical description of the physiology created by a combination of anatomic malformations now referred to as tetralogy of Fallot. The cardinal features (Figure 1) consist of an interventricular communication, or ventricular septal defect, biventricular connection of the aortic root, which overrides the muscular ventricular septum, obstruction of the right ventricular outflow tract, and right ventricular hypertrophy [2]. Each component can vary in its severity, with the variation directly affecting the manifestation and management of the disease.

## Epidemiology

Tetralogy of Fallot occurs in 3 of every 10,000 live births. It is the commonest cause of cyanotic cardiac disease in patients beyond the neonatal age, and accounts for up to one-tenth of all congenital cardiac lesions [3].

## Anatomy

The embryological basis of the combination of lesions is antero-cephalad deviation of the developing outlet ventricular septum, or its fibrous remnant should this septum fail to muscularise. Such deviation, however, can be found in the absence of subpulmonary obstruction, as in the so-called Eisenmenger ventricular septal defect [4]. So as to produce the features of tetralogy of Fallot, therefore, it is also necessary to have abnormal morphology of the septoparietal trabeculations that encircle the subpulmonary outflow tract [5]. The combination of the deviated outlet septum and the hypertrophied septoparietal trabeculations produce the characteristic right ventricular outflow tract obstruction of tetralogy of Fallot (Figure 1). The deviation of the muscular outlet septum is also responsible for creating the malalignment type ventricular septal defect, and results in the aortic override. The associated hypertrophy of the right ventricular myocardium is the haemodynamic consequence of the anatomical lesions created by the deviated outlet septum.

### The ventricular septal defect

**Figure 2:**
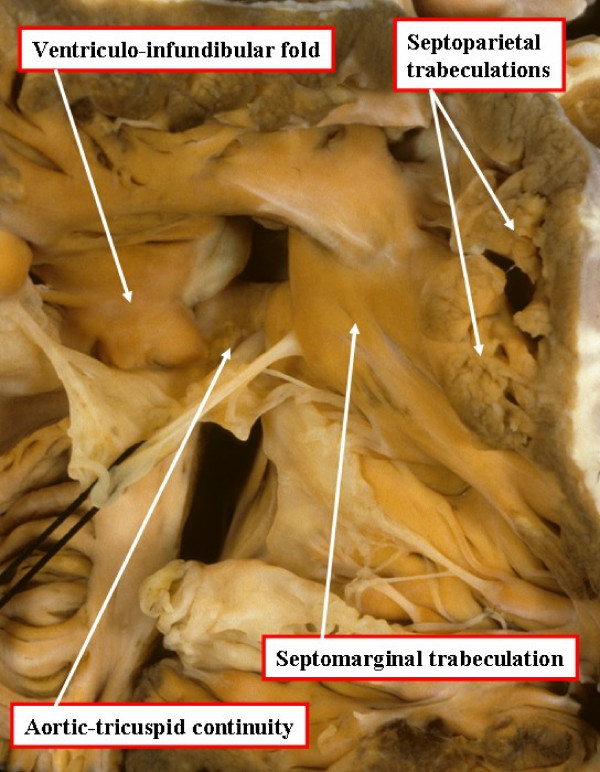
**In this specimen, opened in the same fashion as the heart shown in Figure 1, there is fibrous continuity between the leaflets of the aortic and tricuspid valves in the postero-inferior margin of the ventricular septal defect, making the defect itself perimembranous**.

**Figure 3:**
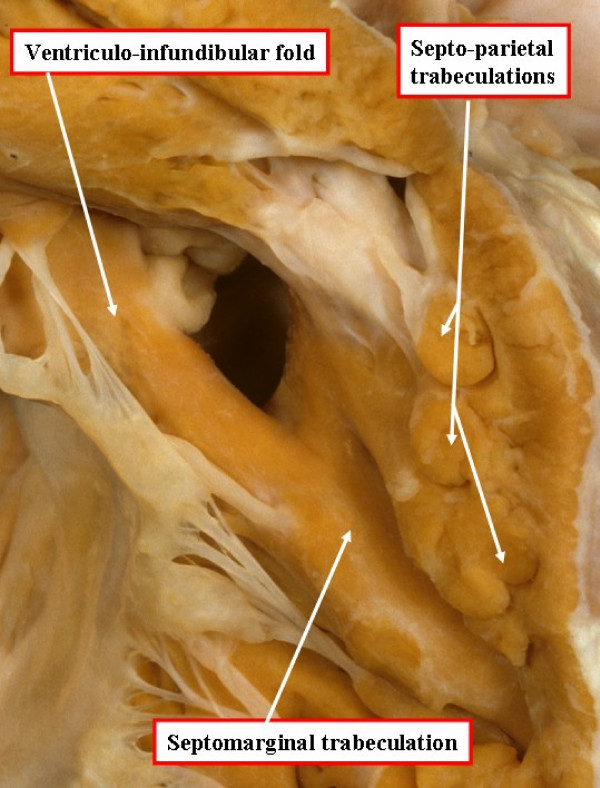
**In this specimen, also photographed in a fashion comparable for the heart shown in Figure 1, there is muscular tissue interposed between the leaflets of the aortic and tricuspid valves in the postero-inferior margin of the ventricular septal defect**.

As discussed above, the interventricular communication found in tetralogy of Fallot exists because of the anterior and cephalad malalignment of the outlet portion of the muscular ventricular septum, or of its fibrous remnant should the outflow cushions fail to muscularise during embryonic development. The resulting hole is one of a number of those appropriately described as a malalignment defect. In four-fifths of Caucasians with such a defect, the postero-inferior margin of the hole between the ventricles is formed by an area of fibrous continuity between the leaflets of the aortic and tricuspid valves, also involving the remnant of the interventricular portion of the membranous septum [5]. In these patients, therefore, the defect is also appropriately classified as being perimembranous (Figure 2). In the remaining one-fifth of Caucasian patients, the postero-inferior rim of the defect is muscular. The muscular bar is formed by continuity of the postero-inferior limb of the septomarginal trabeculation with the ventriculo-infundibular fold (Figure 3). The muscular structure thus formed protects the ventricular conduction axis during surgical closure of the defect.

**Figure 4:**
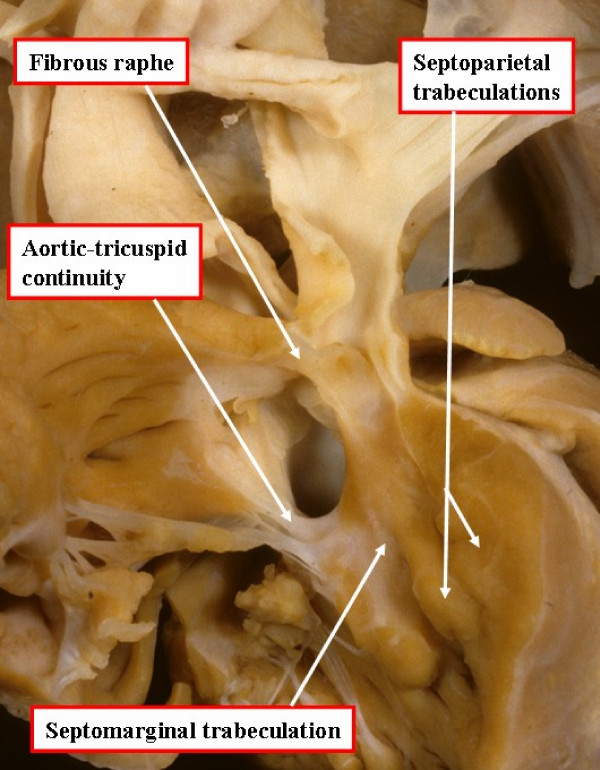
**In this specimen, again photographed as for Figure 1, the defect extends to the level of the pulmonary valve due to failure of muscularisation of the outlet septum during development of the heart**. This type of defect is doubly committed and juxtaarterial, but is also perimembranous.

In a small proportion of Caucasian patients, but in larger numbers of patients seen in the Far East, and in Central and South America, the antero-superior margin of the ventricular septal defect is not formed by the muscular outlet septum, but is formed by fibrous continuity between the leaflets of the aortic and pulmonary valves. This morphology is the consequence of failure of muscularisation of the developing outlet septum (Figure 4). These patients, nonetheless, do have obstruction of the right ventricular outflow tract due to the malalignment of the fibrous remnant of the outlet septum.

The size of the ventricular septal defect can vary, but in almost all instances, the interventricular communication is unrestrictive, allowing for bidirectional shunting [6].

### Override of the aorta

Because of the displacement of the malaligned outlet septum into the right ventricle, the aortic root, of necessity, overrides the muscular ventricular septum. In the setting of significant subpulmonary obstruction, shunting across the interventricular communication is predominantly from right-to-left, which promotes ejection of deoxygenated blood into the systemic circulation. The chronic volume load sustained by the overriding aorta is implicated in the dilation of the aortic root noted in adults with tetralogy of Fallot [7,8].

### Sub-pulmonary obstruction

The antero-cephalad deviation of the outlet septum, coupled with an anomalous relationship to the septoparietal trabeculations, results in a narrowing of the subpulmonary outflow tract. The obstructive muscular subpulmonary area thus created is a dynamic entity. The degree of stenosis created can be exacerbated by catecholamines, or a state of low intravascular volume, predisposing the patients to sudden and acute episodes of desaturation known as hypercyanotic spells [9,10].

The obstruction to flow into the lungs often extends beyond the subpulmonary outflow tract itself. The pulmonary valve may be hypoplastic, with abnormally functioning leaflets, often having a bifoliate configuration. Not infrequently, the pulmonary trunk, and the right and left pulmonary arteries, are diminutive, exhibiting additional focal areas of narrowing [11].

## Anatomical variants of Tetralogy of Fallot, and associated anomalies

### Tetralogy of Fallot with pulmonary atresia

**Figure 5:**
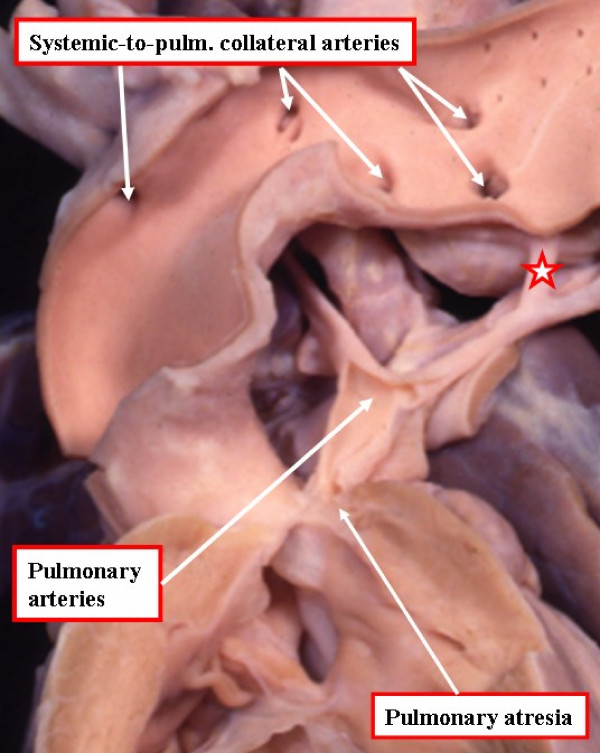
**This specimen has tetralogy of Fallot with pulmonary atresia**. The pulmonary supply is through multiple systemic-to-pulmonary collateral arteries. The star shows the connection between one of the collateral arteries and the intrapericardial pulmonary arteries. All the other arteries join with the intrapericardial pulmonary arterial supply, or else supply segments of the lung directly. The task of the clinician is to display the supply of the various collateral arteries and their communications with the intrapericardial pulmonary arteries.

This lesion is at the most severe end of the spectrum of antero-cephalad deviation of the outlet septum. Occasionally, however, the pulmonary valve is affected in isolation, being imperforate rather than stenotic. In approximately half of patients with pulmonary atresia, the right and left pulmonary arteries are confluent, with blood to the pulmonary arteries flowing through the persistently patent arterial duct. In the other half, the pulmonary arterial supply is multifocal. In these patients, if the pulmonary arteries are confluent or continuous, the blood supply will likely originate only from multiple aorto-pulmonary collateral arteries (Figure 5). If the pulmonary arteries are discontinous or absent, the blood supply to the lungs will originate from multiple collateral arteries, or from a combination of collateral arteries and an arterial duct. It is a general rule that a pulmonary segment will not be supplied by both an arterial duct and a collateral artery [6]. In cases of complex supply of blood to the lungs, it is necessary to determine the proportion of pulmonary parenchyma supplied by the intrapericardial pulmonary arteries as opposed to those parts supplied exclusively by the collateral arteries [6,11,12].

Although the collateral arteries do not depend on prostaglandin for patency, they have the potential to stenose over time. In other instances, large collateral arteries can provide unrestricted flow to the lungs, thus producing hypertensive pulmonary vasculature. The long-term management of the pulmonary supply in patients with tetralogy of Fallot and pulmonary atresia, therefore, is complicated.

### Tetralogy of Fallot with absent pulmonary valve

Malalignment of the outlet septum with rudimentary formation of the leaflets of the pulmonary valve, so-called absent pulmonary valve syndrome, is seen in around one-twentieth of those alleged to have tetralogy of Fallot [13]. The presence of rudimentary valvar leaflets arrayed in circular fashion at the ventriculo-pulmonary junction results in free pulmonary regurgitation throughout foetal life. The end result is that the chronic volume load of the right ventricle is transmitted to the pulmonary arteries, with concomitant dilation of these vessels. In severe cases, patients present with inspiratory and expiratory stridor due to compression of the airways by the dilated pulmonary arteries. Although compression and obstruction of the airways are partly responsible for cyanosis, there is also focal narrowing at the ventriculo-pulmonary junction, contributing to the hypoxaemia in these patients [12,14]. In most instances, but certainly not all, the arterial duct is also absent.

### Tetralogy of Fallot with double outlet right ventricle

With pronounced aortic override, the aorta becomes more committed to the right ventricle than to the left ventricle, resulting in many instances in the ventriculo-arterial connection of double outlet right ventricle. Although the physiology on presentation may not be altered, there are important implications for surgical repair [15]. Patients with the aorta originating predominantly from the right ventricle are at greater risk of developing obstruction to the newly created left ventricular outflow tract, the latter produced by the patch which closes the ventricular septal defect while tunneling the left ventricle to the aorta. This patch, of necessity, is appreciably longer than when the aorta arises mostly from the left ventricle [5,15].

### Tetralogy of Fallot with atrioventricular septal defect

An atrioventricular septal defect combined with a common atrioventricular junction is found in 2% of patients with tetralogy of Fallot [13]. The presentation and initial medical management remain unchanged, but surgical repair and post-operative care are more complex (see Management, [16,17]).

### Associated anomalies

Anomalous origins of the coronary arteries occur in up to one-sixth of patients, and should be documented prior to surgical repair. The most common and relevant anomaly is origin of the left anterior descending artery from the right coronary artery, with the anomalous artery then coursing anterior to the subpulmonary outflow tract, a potential site of surgical incision.

Other associated lesions include atrial septal defects, and additional ventricular septal defects, the latter usually being muscular. Straddling and overriding of the tricuspid valve may also occur, which will complicate the closure of the ventricular septal defect. An important finding when there is overriding of the orifice of the tricuspid valve is the anomalous location of the atrioventricular conduction tissues [18]. A right aortic arch, which is of no haemodynamic consequence, is present in one-quarter of patients with tetralogy of Fallot.

## Clinical manifestations and diagnostic work-up

The initial presentation of tetralogy of Fallot varies depending on the severity of the obstruction of blood flow to the lungs. Most patients will present in the neonatal period with mild-to-moderate cyanosis, but typically without respiratory distress. More uncommonly, patients with very mild right ventricular outflow tract obstruction at birth may be diagnosed at a couple months of age as the obstruction worsens resulting in newly noticed cyanosis and a louder murmur. Because patients with tetralogy of Fallot have obstruction to pulmonary blood flow, they will not present with signs of heart failure such as failure to thrive. Irritability and lethargy are rarely seen in patients with tetralogy of Fallot except in the setting of a hypercyanotic spell. Clubbing is also highly unusual in the modern era since newly diagnosed patients undergo surgical repair before clubbing has time to develop.

The second heart sound in patients with tetralogy of Fallot may be single and loud, and a harsh systolic ejection murmur will be present, emanating from the obstructed subpulmonary outflow tract. Flow across the interventricular communication in tetralogy of Fallot is usually not turbulent, and therefore not audible. Patients with severe obstruction, and very little antegrade flow across the subpulmonary outflow tract, will be more significantly cyanotic and have a less prominent murmur.

Once the lesion is suspected, an electrocardiogram and chest radiograph should be performed. The electrocardiogram will demonstrate right axis deviation and prominent right ventricular forces, with large R waves in the anterior precordial leads and large S waves in the lateral precordial leads [19]. Although the electrocardiogram is similar to that of a normal newborn, the right ventricular hypertrophy and right axis deviation will not normalize in a patient with tetralogy of Fallot. The classical chest radiograph will demonstrate a boot-shaped cardiac silhouette. This is due to upward displacement of the right ventricular apex as a consequence of the right ventricular hypertrophy, and a narrowing of the mediastinal shadow due to the hypoplastic pulmonary outflow tract.

**Figure 6:**
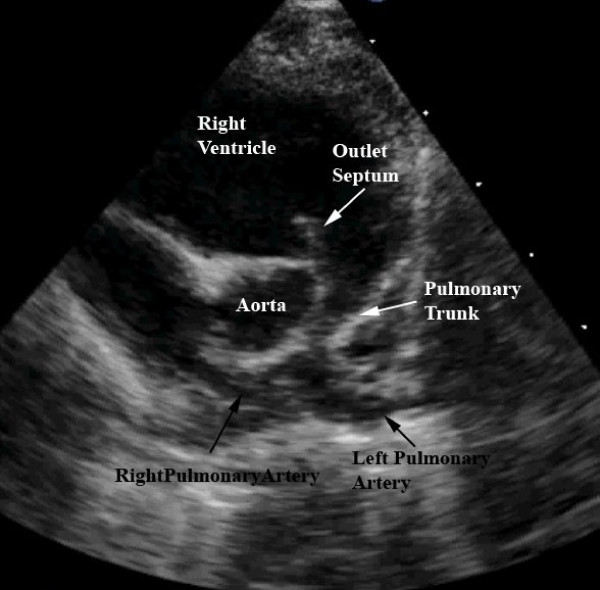
**This still frame image of a parasternal short axis view of the echocardiogram of a patient with tetralogy of Fallot demonstrates the antero-cephalad deviation of the outlet septum into the right ventricular outflow tract**.

**Figure 7:**
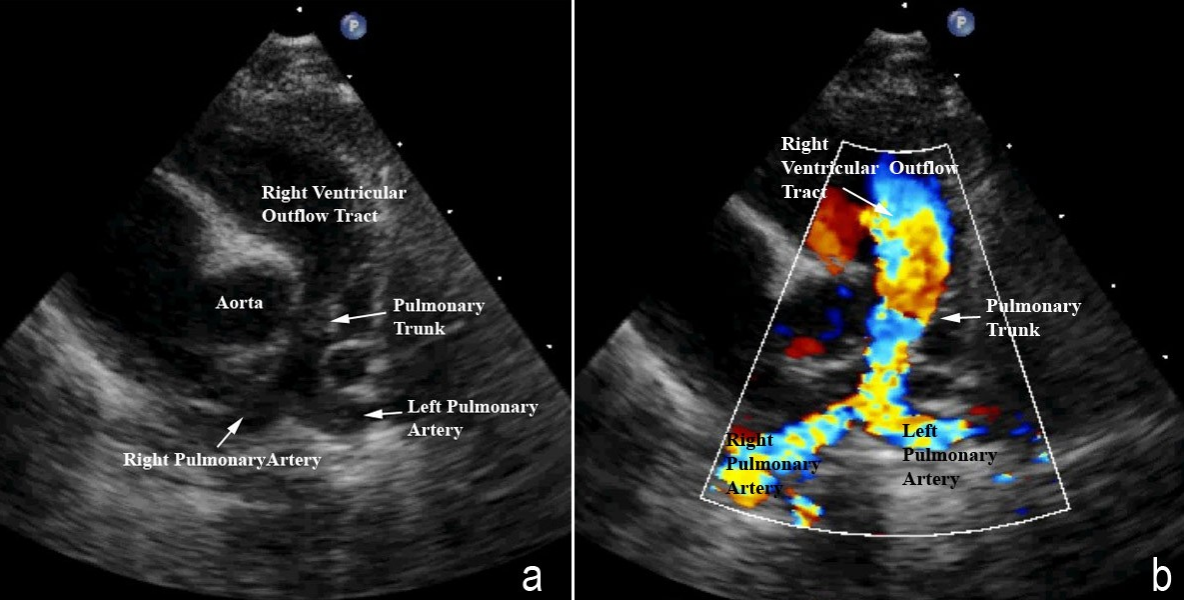
**A slightly modified view (a), angled to optimize imaging of the pulmonary arteries in the patient imaged to produce Figure 6, reveals significant hypoplasia of the pulmonary trunk and the pulmonary arteries, which result from the antero-cephalad deviation of the outlet septum**. The pulmonary valvar leaflets are not visualized. In panel b, colour Doppler has been used, and demonstrates turbulence and acceleration of the flow of blood in the right ventricular outflow tract, originating at the level of the deviated outlet septum. The turbulence continues into the hypoplastic pulmonary trunk and pulmonary arteries.

**Figure 8:**
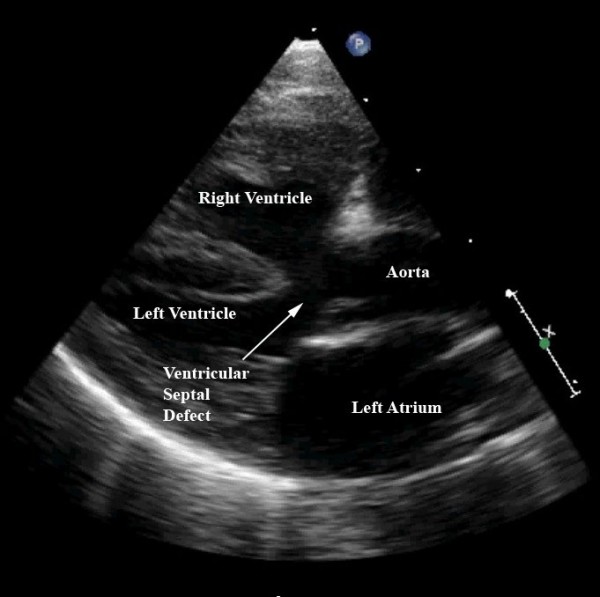
**This still frame of a modified parasternal long axis view from the same patient as imaged for Figures 6 and 7 demonstrates the large ventricular septal defect, aortic override, and right ventricular hypertrophy charactistic of patients with tetralogy of Fallot**.

Diagnosis is confirmed with echocardiography. The severity of the subpulmonary obstruction, its dynamic component, the size of the right and left pulmonary arteries, and any additional sources of flow of blood to the lungs will all be delineated (Figures 6, 7a, 7b). The degree of aortic override, the size of the interventricular communication, as well as the presence of other associated lesions, will be identified (Figure 8). Cardiac catheterisation is now rarely needed due to the high sensitivity and specificity of echocardiographic images.

### Hypercyanotic spells

The hypercyanotic spell [9,10] is characterised by a sudden and striking decrease in the saturation of oxygen due to acute and complete, or near complete, obstruction of the subpulmonary outflow tract. Not all patients with tetralogy of Fallot will have hypercyanotic spells. The spells typically begin to occur at approximately a couple months of age at times of agitation or decreased hydration, both of which exacerbate the dynamic infundibular obstruction. The murmur produced by the muscular obstruction is absent during a true spell, due to nearly absent antegrade flow across the right ventricular outflow tract. Patients become severely cyanotic, hyperpnoeic, and lethargic. With the development of metabolic acidosis, there is an increase in pulmonary vascular resistance, with a decrease in systemic vascular resistance. Cardiac output becomes compromised due to myocardial ischaemia. Impending collapse and death can ensue.

## Aetiology and genetic counselling

The aetiology is multifactorial. Associated chromosomal anomalies can include trisomies 21, 18, and 13, but recent experience points to the much more frequent association of microdeletions of chromosome 22. As many as one-eighth of patients will have chromosomal abnormalities, such as trisomy 21, 18, or 13 [3]. Up to one-fifth of patients with tetralogy and pulmonary stenosis, and two-fifths of those with tetralogy and pulmonary atresia, will have microdeletions of chromosome 22q11.2 [12,20,21]. The deletion, manifested by varying degrees of palatal abnormalities, dysmorphic facies, learning disabilities, immune deficiencies, and hypocalcaemia, is frequently referred to as the DiGeorge Syndrome.

The risk of recurrence in a family is approximately 3%.

Associations with maternal intake of retinoic acid during the first trimester, poorly controlled diabetes, and untreated phenylketonuria have also been described [11].

## Antenatal diagnosis

Tetralogy of Fallot can be diagnosed antenatally as early as 12 weeks of gestation [22]. In a population-based study, however, only half of the cases were detected during routine obstetric ultrasonic screening [23]. In general, patients who are referred for foetal echocardiography with a suspicion of tetralogy of Fallot have the most severe phenotypes [23]. Other reasons for referral for foetal echocardiography include discovery of extra-cardiac malformations, or known chromosomal abnormalities. As a result, patients referred for foetal echocardiography tend to have worse outcomes when compared to patients who are diagnosed postnatally [23]. The foetus with tetralogy can be delivered vaginally, but efforts should be made for delivery to occur in a centre where paediatric cardiologists are available to aid in the postnatal care.

## Differential diagnosis

The differential diagnosis of any cyanotic patient with a murmur will include persistent pulmonary hypertension of the newborn, as well as other cyanotic lesions such as critical pulmonary stenosis, Ebstein's malformation, transposed arterial trunks, common arterial trunk, totally anomalous pulmonary venous connection, and tricuspid atresia.

## Management

Clinical management is determined by the degree and type of subpulmonary obstruction, in combination with the preference of the centre for the timing of surgical intervention. Depending on the severity of obstruction within the subpulmonary outflow tract, an infusion of prostaglandin may be initiated to preserve ductal patency, and provide a stable source of flow of blood to the lungs. Patients who require such an infusion will most likely require surgical intervention prior to discharge from the hospital. For those patients who have adequate forward flow through the subpulmonary outflow tract after ductal closure, management consists of close follow-up until a complete repair is performed.

Some centres will perform complete repairs in all neonates. Others will palliate symptomatic neonates, and perform a complete repair in all patients at the age of 4 to 6 months.

Palliation, which frequently does not require cardiopulmonary bypass, establishes a secure source of flow of blood to the lungs by placing a prosthetic tube between a systemic and a pulmonary artery. The most common type of aorto-pulmonary shunt is known as the modified Blalock-Taussig shunt. This consists of a communication between a subclavian and pulmonary artery on the same side. A complete repair, always performed under cardiopulmonary bypass, consists of closing the interventricular communication with a patch channeling the left ventricle to the aortic root, relief of the subpulmonary obstruction, and reconstruction, if necessary, of the pulmonary arteries.

Complete neonatal repair provides prompt relief of the volume and pressure overload on the right ventricle, minimises cyanosis, decreases parental anxiety, and eliminates the theoretical risk of stenosis occurring in a pulmonary artery due to a palliative procedure. Patients who undergo a successful complete repair during the neonatal period will be unlikely to require more than one intervention in the first year of life, but are not free from reintervention. Concerns regarding such neonatal complete repairs include exposure of the neonatal brain to cardiopulmonary bypass, and the increased need to place a patch across the ventriculo-pulmonary junction when compared to older age at repair [24,25]. Patches placed across the ventriculo-pulmonary junction, so-called transannular patches, create a state of chronic pulmonary regurgitation, which increases morbidity in young adults, producing exercise intolerance and ventricular arrhythmias. If left untreated, this increases the risk of sudden death [26-28]. The effect of cardiopulmonary bypass on the neonatal brain, and the associated risk of longer stay in hospital and the intensive care unit, is not trivial. Studies of neurodevelopmental outcomes of neonates undergoing cardiopulmonary bypass compared to older children have shown lower intelligence quotients in patients exposed to bypass as neonates [29]. Longer periods of bypass, and longer stays in the intensive care unit, have been associated with an increased risk for neurological events and abnormal neurological findings on follow-up [30,31]. While some studies have not found cyanosis itself to be responsible for cognitive problems in children with congenitally malformed hearts [32], others have implicated chronic cyanosis as a factor contributing to impaired motor skills, decreased academic achievement, and worsened behavioural outcomes [33,34]. In the absence of randomised control trials, assessing the risk and benefits of the two surgical strategies has been notoriously difficult. 

In summary, patients with cyanotic tetralogy of Fallot will either undergo neonatal complete repair or neonatal palliation with an aortopulmonary shunt followed by a complete repair at four to six months of age. Peri-operative mortality rates for either surgical approach is less than 3% [24,25] and since neither strategy has shown superior results, surgical management remains dependent on the protocols preferred by the individual centres.

### Management of the anatomical variants of tetralogy of Fallot

Neonates born with tetralogy of Fallot with pulmonary atresia who do not have stable flow to the lungs through collateral arteries will require surgical intervention prior to discharge from the hospital. In newborns with tetralogy of Fallot with absent pulmonary valve, surgical repair may be required prior to initial discharge from hospital if obstruction to the airways is a prominent symptom. In cases with tetralogy of Fallot with atrioventricular septal defect, complete repair is usually performed later in life compared to patients with uncomplicated tetralogy of Fallot, typically between the ages of 6 and 12 months. If significantly cyanotic at birth, most centres still opt for initial palliation rather than complete repair in these patients. Freedom from reoperation is decreased compared to patients with uncomplicated tetralogy of Fallot [17].

### Management of the hypercyanotic spell

Overcoming a hypercyanotic spell requires maneuvers to re-establish adequate balance between the systemic and pulmonary flows. Treatment must focus on decreasing pulmonary, and increasing systemic, vascular resistance, hence promoting left to right flow across the ventricular septal defect and into the subpulmonary outlet.

Parents at home with a child suffering such spells are taught to place their child in the knee-to-chest position in an effort to increase systemic vascular resistance and promote systemic venous return to the right heart. This will theoretically increase intracardiac shunting from left-to-right across the interventricular communication, as well as increase the preload of the right ventricle. Emergency services should be contacted immediately.

Medical management will consist of establishing immediate intravenous access to allow prompt administration of fluids, which will improve right ventricular preload. Oxygen should be initiated to decrease peripheral pulmonary vasoconstriction, and improve oxygenation once flow of blood to the lungs is re-established. Subcutaneous morphine should be administered to decrease the release of catecholamines. This will increase the period of right ventricular filling by decreasing the heart rate, and promote relaxation of the infundibular spasm. If the patient remains hypercyanotic after these measures, he or she should be paralysed and intubated, with phenylephrine administered intravenously to increase systemic vascular resistance.

The long half-life, and potential side effects, such as hypotension and cardiac dysfunction, of beta blockers precludes their routine use in the emergency situation. Propranolol has been used in small doses in the chronic care of patients deemed to be at risk for spells in an effort to minimise the infundibular spasm responsible for the episodes. Once a patient requires prophylaxis by beta-blockade, surgical referral should occur to prevent the potential tragic and unpredictable outcome of a hypercyanotic spell [10].

## Prognosis

Studies of immediate and long-term follow-up in tetralogy of Fallot reveal excellent outcomes. Patients born 30 years ago with tetralogy of Fallot have an 85% long-term rate of survival, and in the absence of serious residuae are able to lead normal lives as evidenced by the ability to carry successful pregnancies for example [35-40]. Chronic issues that face the current population of adults subsequent to their surgical repair include the haemodynamic manifestations of chronic pulmonary regurgitation, recurrent or residual pulmonary stenosis, and ventricular arrhythmias. The prognosis of patients born in the current era is expected to be substantially improved due to advances in surgical and medical management that have occurred over the past couple of decades. As for all patients with congenitally malformed hearts, the management of the patient with tetralogy of Fallot does not end at the time of complete repair. Follow-up by cardiologists trained in congenital cardiac disease will remain a life-long experience.

## Unresolved questions

**Figure 9:**
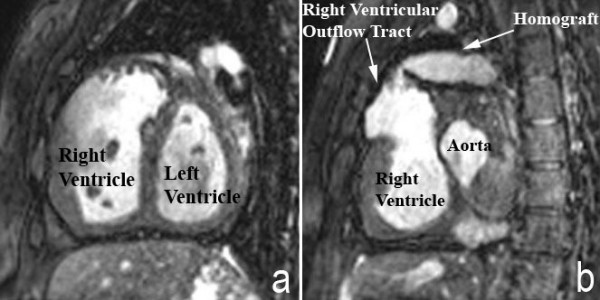
**Cardiac magnetic resonance imaging in an adult patient with tetralogy of Fallot repaired in childhood with an infundibular muscle resection and placement of a conduit from the right ventricle to the pulmonary arteries (a) has results in dilation and hypertrophy of the right ventricle**. Additional images (b) show the stenotic and tortuous homograft conduit responsible for the dilated and hypertrophied right ventricle.

Questions that remain regarding the management of tetralogy of Fallot pertain to the preference of surgical timing, as discussed above, and to the residual lesions of the disease. The management of young adults with pulmonary regurgitation or residual pulmonary stenosis is complex. A balance must be achieved between limiting the haemodynamic burden on the right ventricle, and minimizing the life-time number of surgical interventions required for a given patient. Cardiac magnetic resonance imaging has progressed significantly in the past ten years (Figures 9a and 9b). This provides reliable objective data, in addition to echocardiography and exercise testing, that physicians can use to determine the optimal timing of reintervention [41-43]. Recent advances in interventional cardiac catheterisation now provide safe options for treatment other than cardiac surgery. Devices such as the percutaneously placed pulmonary valve have shown promising results in the management of selected patients with pulmonary regurgitation and stenosis [44,45]. Ongoing advances in diagnostics and minimally invasive procedures will continue to help improve the care of these adult patients with tetralogy of Fallot.
